# Buccal bone thickness at dental implants in the maxillary anterior region with large bony defects at time of immediate implant placement: A 1‐year cohort study

**DOI:** 10.1111/cid.12701

**Published:** 2018-12-11

**Authors:** Henny J. A. Meijer, Kirsten W. Slagter, Arjan Vissink, Gerry M. Raghoebar

**Affiliations:** ^1^ Department of Implant Dentistry University of Groningen, University Medical Center Groningen Groningen The Netherlands; ^2^ Department of Oral and Maxillofacial Surgery University of Groningen, University Medical Center Groningen Groningen The Netherlands

**Keywords:** bone thickness, cone‐beam computed tomography, dental implants, esthetic region

## Abstract

**Background:**

There is lack of studies regarding preservation and possible changes in BBT at dental implants.

**Purpose:**

To assess, on cone beam computer tomograms, the presence of bone at the time of tooth extraction in the maxillary esthetic region and the mean buccal bone thickness 1 month and 1 year after final restoration placement in patients with large bony defects.

**Material and Methods:**

In a cohort study, patients were selected presenting a failing tooth with a large bony defect (test group [*n* = 20]: *large bony defect*, immediate placed implant and delayed provisionalization). Results were compared with a group in which patients presented a failing tooth without or with a small bony defect: (control group [*n* = 20]: *without or small bony defect*, immediate placed implant and delayed provisionalization). Cone beam computer tomograms were made preoperatively, and 1 month and 1 year after placement of the restoration, and buccal bone thickness was analyzed.

**Results:**

In both groups approximately 1 mm of buccal bone thickness was present after 1 month and 1 year, without a significant difference between the groups.

**Conclusion:**

In patients with large bony defects at a failing tooth it was possible to create a bone layer buccally of the implant and this bone layer remained stable during a 1‐year follow‐up; there were no significant differences between thickness of buccal bone at 1 month and 1 year in patients with large buccal bony defects and patients without or with small bony defects.

## 1 INTRODUCTION

Immediate implant placement in the esthetic region is stated to be a favorable treatment modality for replacing failing teeth.^1^, ^2^, ^3^ Conversely, there are also studies which conclude to be cautious with immediate implant placement, especially if highly esthetic demands are involved.^4^ Particularly in the esthetic zone, preservation and establishment of labial mucosa and underlying buccal bone has been shown to be a key factor in achieving optimal results.^5^, ^6^

In the esthetic region, there is data regarding buccal bone thickness (BBT) when the tooth is still in situ.^7^, ^8^ Januário and colleagues^7^ showed that the buccal bone wall in most cases was less than 1.0 mm thick and even in half of the sites 0.5 mm or less. In the study of El Nahass and Naiem^8^ it was reported that usually mean BBT at the central and lateral incisors was less than 1.0 mm. Removal of a maxillary anterior tooth will lead to significant loss of BBT within a few weeks.^9^ It has been posed that immediate dental implant placement with augmentation of the space between implant and buccal wall should prevent these dimensional changes, but the results of this preservation technique are contradictory.^3^, ^10^, ^11^ It is of interest if the original buccal wall still exists in time or disappears and the augmented bone functions as a new buccal plate at the implant.^12^

If a large bony defect results after extraction, hard and soft tissue grafting is often recommended in combination with delayed implant placement.^13^, ^14^ However, there are also studies reporting a favorable esthetic outcome when placing implants in fresh extraction sockets with buccal wall dehiscences.^15^, ^16^

The morphological assessment of buccal bone volume before placement of dental implants and at dental implants during a follow‐up period is of great interest to clinicians to predict reliability of treatment in the esthetic region. Cone‐beam computed tomography (CBCT) has been successfully used for various dental procedures.^17^ The CBCT has also been used to assess buccal bone dimensions prior and after implant placement.^18^, ^19^

Despite the interest to clinicians, there is lack of studies regarding preservation and possible changes in BBT at dental implants, especially with large bony defects at the time of immediate implant placement. One reason is because in analyzing BBT on CBCT's difficulties are encountered with standardization of measurements. The use of three‐dimensional (3D) image diagnostic and treatment planning software programs could be helpful.^20^

The purpose of the present cohort study was to assess, on CBCT's, BBT at the time of tooth extraction in the esthetic region in patients with large bony defects and 1 month and 1 year after final restoration placement and compare it with a group without or with small bony defects.

## 2 MATERIAL AND METHODS

### 2.1 Patient selection

Forty participants with an implant‐supported restoration in the esthetic region of the maxilla were included in the study, originally part of two randomized controlled trials performed at the University Medical Center Groningen in the Netherlands.^21^, ^22^ These trials got approval by the Medical Ethic Board (METC 2010.246) and registered (www.isrtcn.com: ISRCTN57251089). All participants gave written informed consent and research was carried out in accordance with the Declaration of Helsinki.

The following group was selected from a study in which patients presented a failing tooth in the maxillary esthetic region with a large bony buccal defect^21^:

- test group (*n* = 20): *large bony defect*

and compared with a group selected from a study in which patients presented a failing tooth in the maxillary esthetic region without or with a small bony buccal defect^22^:

- control group (*n* = 20): *without or small bony defect*

A large bony defect was defined as being ≥2 mm and a small bony defect as <2 mm, after the review of Chen and colleagues^23^ and confirmed by the Consensus Statements of Hämmerle and colleagues.^24^ In both the test group and the control group, implants were immediately placed implant and delayed provisionalized.

Characteristics at baseline of the study groups are:

- test group (*n* = 20): mean age in years (range): 43.7 (18‐63); male/female: 11/9; location of implants (central incisor/lateral incisor/canine): 12/5/3;
- control group (*n* = 20): mean age in years (range): 42.3 (23‐66); male/female: 8/12; location of implants (central incisor/lateral incisor/canine): 13/6/1.

**Figure 1:**
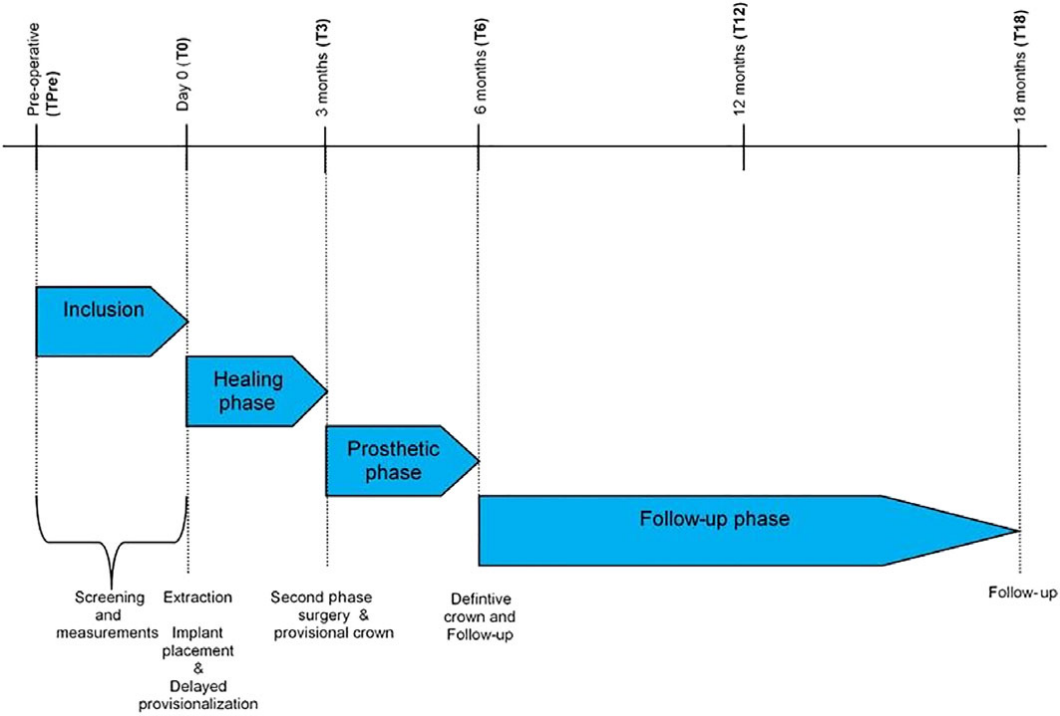
Schedule of visits and procedures test group and control group

The time path for both test and control group is illustrated in Figure 1. For details with regard of selection of the patients, allocation to the groups, surgical and prosthetic procedures and analysis performed see Slagter and colleagues.^21^, ^22^ A short description is presented below.

### 2.2 Surgical and prosthetic procedures

Surgical procedures were performed by the same experienced oral and maxillofacial surgeon.

Failing teeth were removed with a sulcular incision, careful detachment of the periodontal ligament, and use of periotomes, without flap elevation. After removal of the tooth, the alveolus was meticulously cleansed, and any alveolar debridement was removed with sterile gauze. Before implant placement, bone grafts were harvested from the maxillary tuberosity with the use of chisels. The implant site was prepared on the palatal side of the alveolus according to manufacturer protocol using a surgical template for ideal positioning of the prospective implant crown. The bur used last, depending on diameter of implant, was placed in the prepared alveolus. Next, the tuberosity bone graft was shaped with forceps to match the labial bony defect. The bone graft was placed in the extraction socket, with the cortical side facing the periosteum, under the periosteum covering the labial plate defect. A mixture of autologous bone and Bio‐Oss spongiosa granules (0.25‐1.0 mm, Geistlich, Wolhusen, Switzerland) was tightly packed into remaining space. The implant (NobelActive, Nobel Biocare AB, Goteborg, Sweden) was placed at a depth of 3 mm apical to the most apical aspect of the prospective clinical crown, with the help of the surgical template. Immediately after implant placement, a corresponding cover screw was placed. No membranes were used to cover the grafted area. To achieve an optimal esthetic outcome, a soft tissue graft, harvested from the tuberosity region where the bone graft was taken, was placed on top of the bone graft and implant. The wound was closed with 5‐0 nylon sutures. During the 3‐month osseointegration phase, patients were allowed to wear a removable partial denture not interfering with the wound. After 3 months, the implant was uncovered by a small incision at the cover screw site, followed by an implant‐level impression. Within 8 hours, a screw‐retained provisional restoration was placed. After a provisional phase of 12 weeks a final restoration was placed. Prosthetic procedures were performed by a single prosthodontist.

In the control group (with a small bony defect), the same surgical procedures were performed, with the exception that no bone graft from the maxillary tuberosity was harvested. Only a mixture of autologous bone and Bio‐Oss spongiosa granules (0.25‐1.0 mm) was used to fill the remaining space between implant and buccal bony wall.

### 2.3 CBCT measuring procedure

To define the presence and thickness of bone at the time of tooth extraction and to measure changes in the BBT over time, CBCT's (iCAT 3D exam scanner, KaVo Dental GmbH, Biberach, Germany) were made before extraction and after 1 month and 1 year after placement of the final restoration. This scanner was validated for measuring bone thickness by Fourie and colleagues.^25^ They reported a method error of only 0.05 mm (95 CI 0.03‐0.07 mm). Bone thickness measurements were done using 3D image diagnostic and treatment planning software (NobelClinician, version 2.1, Nobel Biocare ‐ Guided Surgery Center, Mechelen, Belgium). A CBCT imaging and software protocol, developed and validated by Slagter and colleagues, was used.^20^

Of each patient, the position of the implant was determined by importing the 1‐month and 1‐year CBCT, in DICOM multi‐file format, into an image computing program, Maxilim, version 2.3 (Medicim, Sint‐Niklass, Belgium). With the concept of multimodality image registration using information theory (MIRIT) the exact position of the implant can be recognized, determined, and implemented in the patients DICOM files.^26^ The MIRIT procedure finds its base on recognizing image similarities. The degree of similarity between intensity patterns in two images (one of the implant with the dimensions used in the patient and the one of the depicted implant in the DICOM file of the patient) is determined, and consequently, the recognized image is registered automatically into one coordinate system.

**Figure 2:**
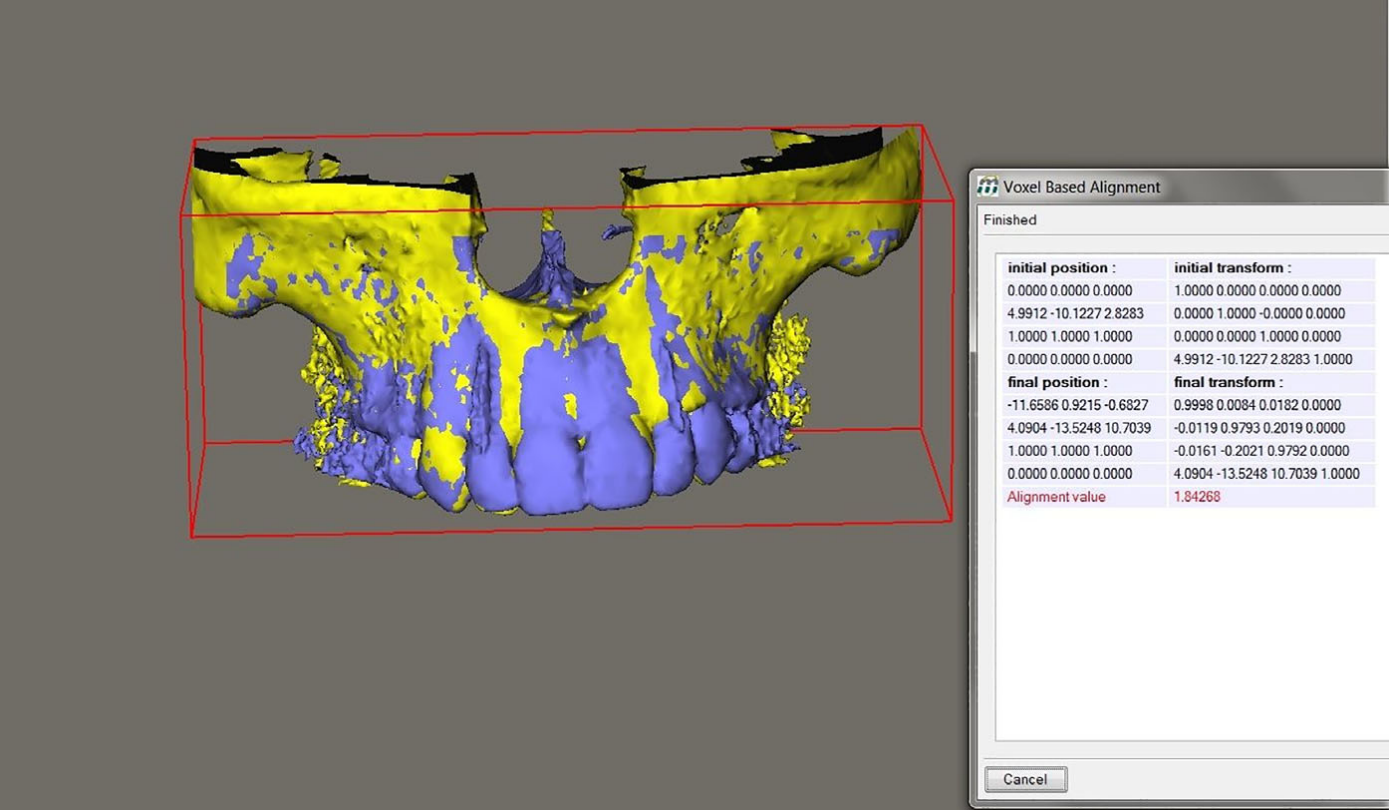
Alignment of images of pretreatment cone‐beam computed tomography (CBCT) and the 1‐month CBCT by the computing program Maxilim

A different procedure was followed for the pretreatment CBCT's in which no implant was present yet and MIRIT cannot recognize an implant position. First, both the pretreatment CBCT and the 1‐month CBCT were imported in Maxilim. Both images were aligned by the computing program. Because the exact position of the implant has been determined for the 1‐month image, it is now possible to implement this position in the pretreatment DICOM file. In this way, a combined file has been constructed in which the tooth is still present and an implant has been imported in the exact position where it is going to be after treatment (Figure 2).

**Figure 3:**
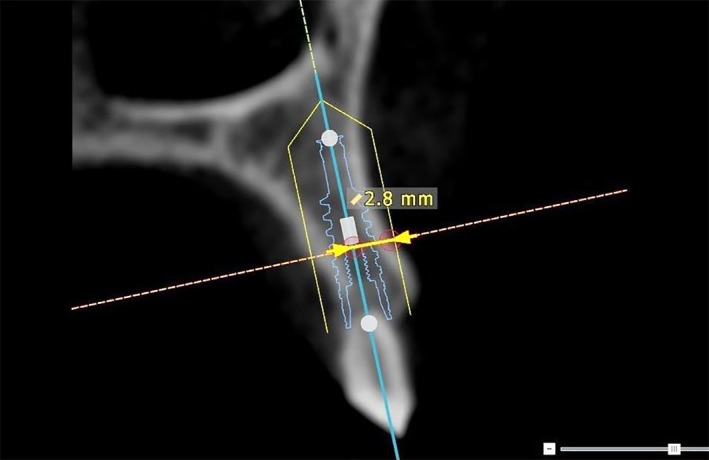
Measurement on a pretreatment cone‐beam computed tomography from the central axis of a planning implant, placed in the position where the actual implant will be after surgery, to the outer surface of the buccal bony wall of a failing natural tooth with the planning program NobelClinician

**Figure 4:**
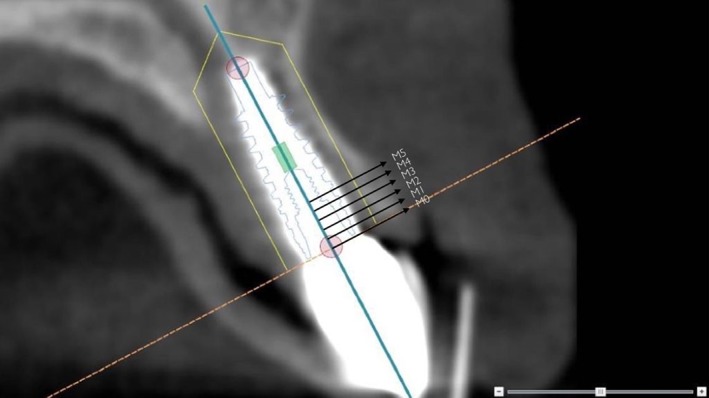
Measurement on a 1‐month cone‐beam computed tomography from the central axis of a planning implant, placed in the position of the actual implant, to the outer surface of the buccal bony wall at the implant with the planning program NobelClinician

In NobelClinician, the exact position of the implant, as determined in Maxilim, was aligned with a planning implant. Buccal bone measurements, at midline of the implant, were performed with the standard provided measurement options of NobelClinician. The upper 5 mm section of the implant was defined as the area of interest, beginning at the neck of the implant. Buccal bone measurements (in mm) were performed from the radius of the interior contour of the implant to the outer surface of the bone. In this way, measuring at the interface between implant and bone, often disturbed by scattering, was avoided. Buccal bone thickness was measured for 5 mm along the axis beginning at the neck of the implant (M0) toward apical (M1, M2, M3, M4, M5; Figures 3 and 4).

### 2.4 Clinical soft tissue outcomes and esthetic appearance

To complete insight of the labial aspect of soft tissues at dental implants, the following clinical and esthetic items have been evaluated:

- change in midfacial mucosal level in mm at 1 year as compared with the gingival level of the preoperative failing tooth;
- pocket probing depth in mm buccally of the implant at 1 year;
- esthetic appearance scored with the pink esthetic score (PES) at 1 year (Belser and colleagues).^27^

### 2.5 Statistical analysis

For comparison between test and control group, the Mann‐Whitney *U* test was performed. A *P‐*value of .05 was considered being statistical significance.

## 3 RESULTS

One patient from the control group did not show up at follow‐up and was excluded from further analysis. Five pretreatment CBCT's were not available (two in the test group and three in the control group). This resulted in CBCT's of 34 patients available for the present study. Median and interquartile ranges, together with means and standard deviations, of bone thickness at M0‐M5 at pretreatment, 1 month and 1 year after final restoration placement are depicted per study group in Table 1.

**Table 1:** Buccal bone measurements pre‐extraction, 1 month and 1 year after dental implant surgery in the test group and control group (test group: large bony and immediate placement/delayed provisionalization; control group: without or small bony defect and immediate placement/delayed provisionalization) expressed as median and mean and significant differences between the groups

Measurements pre‐extraction	Test group (*n* = 18)		Control group (*n* = 16)		
	Median (interquartile range) in mm	Mean (SD) in mm	Median (interquartile range) in mm	Mean (SD) in mm	Significance*
M0 (at neck)	0 [0;0]	0.00 (−)	2.14 [1.73;2.61]	2.20 (0.58)	*P* < .001
M1	0 [0;0]	0.00 (−)	2.21 [1.88;2.59]	2.23 (0.47)	*P* < .001
M2	0 [0;0]	0.00 (−)	2.28 [1.67;2.48]	2.17 (0.48)	*P* < .001
M3	0 [0;0]	0.00 (−)	2.23 [1.75;2.35]	2.08 (0.48)	*P* < .001
M4	0 [0;0.72]	0.51 (0.96)	1.99 [1.75;2.35]	1.98 (0.57)	*P* < .001
M5	0 [0;1.49]	0.68 (0.93)	1.99 [1.56;2.50]	1.93 (0.68)	*P* < .001
Measurements 1 month					
M0 (at neck)	0.89 [0.74;1.17]	1.08 (0.52)	0.94 [0.54;1.98]	1.27 (0.82)	*P* = .878
M1	1.16 [0.66;1.84]	1.30 (0.63)	1.06 [0.51;2.36]	1.39 (0.95)	*P* = .986
M2	1.15 [0.65;1.60]	1.22 (0.63)	1.48 [0.60;2.25]	1.46 (0.82)	*P* = .463
M3	1.25 [0.73;1.78]	1.28 (0.63)	1.34 [0.57;2.01]	1.39 (0.76)	*P* = .695
M4	1.04 [0.71;1.77]	1.27 (0.71)	1.45 [0.45;1.91]	1.32 (0.75)	*P* = .878
M5	0.81 [0.54;1.69]	1.12 (0.68)	1.21 [0.38;1.63]	1.19 (0.70)	*P* = .986
Measurements 1 year					
M0 (at neck)	0.84 [0.64;1.14]	1.01 (0.45)	1.05 [0.57;2.01]	1.24 (0.83)	*P* = .721
M1	1.09 [0.71;1.58]	1.16 (0.57)	1.34 [0.58;2.20]	1.36 (0.79)	*P* = .574
M2	1.05 [0.65;1.63]	1.23 (0.72)	1.43 [0.73;2.28]	1.48 (0.79)	*P* = .403
M3	1.05 [0.55;1.80]	1.26 (0.76)	1.60 [0.65;1.98]	1.39 (0.69)	*P* = .621
M4	0.85 [0.62;1.82]	1.19 (0.74)	1.44 [0.44;1.85]	1.26 (0.70)	*P* = .986
M5	0.86 [0.54;1.69]	1.09 (0.68)	1.31 [0.38;1.63]	1.14 (0.60)	*P* = .932

* Mann‐Whitney *U* test for significant differences between medians of groups at three time points.

In patients from the test group, the median distance from the outer surface of the buccal bone to the surface of the future implant at all different positions was at pretreatment 0 mm. One year after definitive crown placement median BBT varied from 0.84 to 1.09 mm. In patients from the control group, the median distance of six positions from the outer surface of the buccal bone to the surface of the future implant at pretreatment varied from 1.99 to 2.28 mm. One year after definitive crown placement median BBT was more than 1 mm at all six positions. At 1 month and 1 year, there was not a significant difference in BBT between the groups. There is not a significant difference between BBT at 1 month and 1 year in both the test group and the control group.

Clinical soft tissue outcomes and esthetic appearance of both groups are depicted in Table 2.

**Table 2:** Change in midfacial mucosal level (MML) at 1 year as compared with the gingival level of the preoperative failing tooth, pocket probing depth (PPD) buccally of the implant at 1 year and pink esthetic score (PES) at 1 year of the test group (large bony defect) and control group (small bony defect)

	Test group (*n* = 18)	Control group (*n* = 16)
Mean change in MML in mm (SD)	−0.2 (0.3)	−0.8 (0.9)
Mean PPD in mm (SD)	3.2 (0.8)	3.0 (0.6)
Mean PES (SD)	7.5 (1.6)	7.4 (1.5)

## 4 DISCUSSION

In patients with large buccal bone defects at time of immediate implant placement it appeared to be possible to create a new buccal bone plate, which was stable during a 1‐year follow‐up period. Results were not significantly different from patients without or with small bony defects.

In the test group, there was no bone present at the first 3 mm at the labial side of the future implant position. At positions M4 and M5, distance from outer contour of the bone to the surface of the virtual implant was small. The next CBCT in this group was taken after 7 months. At this time point, 1 month after placement of the definitive restoration, median BBT was approximately 1 mm at any position. Between 7 months and 18 months, BBT remained stable without a significant difference between the time periods. This means that the augmented bone functioned as a stable new buccal plate. This outcome can be compared with results of the case series study of Sarnachiaro and colleagues,^16^ in which implants were immediately placed in patients with large buccal defects. At start of the restorative phase, after 6 months of healing, at the neck of the implant a mean BBT of 3.0 mm was achieved. It must be noted that in the present study this was much less, being a little bit more than 1 mm.

In the control group without or with small bony defects, there was always bone present before treatment at the buccal side of the future implant position, varying from a median value from outer contour of the bone to the surface of the virtual implant of 1.99‐2.28 mm. Consistent with the test group, the next CBCT in the control group was taken after 7 months. In these 7 months, the median BBT diminished significantly with at least 0.5 mm. It could well be that the total original wall has been resorbed in these 7 months. Between 7 months and 18 months, there was not a significant difference in BBT between the time periods, meaning that also in this group buccal bone remained stable during the first year of follow up, even when the original buccal wall had been resorbed. This could mean that the newly augmented bone functioned as a new buccal plate. Outcomes in the control group are comparable with results of the study of Mazzocco and colleagues^28^ in which CBCT measurements were presented of a group with immediate implant placement in cases without bony defects. The method used was superimposition of CBCT's. They found a mean diminution of BBT of about 0.6 mm in the first millimeters along the implant axis after 6 months, stating that most of the original buccal wall must have been resorbed. A comparable result was also found in the present control group.

At 7 months and at 18 months, BBT was not significantly different between patients from the test group and the control group at all six evaluated positions along the implant axis and BBT appeared to be stable. This means that in patients with a failing tooth in the maxillary esthetic region immediate implant placement is possible if initial implant stability can be achieved, irrespective of the presence of a buccal bone defect.

It appeared from the present study that buccal bone thickness at dental implants in the esthetic region was hardly subject to change. This can be called a very favorable outcome, because it means that bone thickness achieved at placement of the final restoration remains stable. Moreover, if future evaluation with longer follow‐up evaluations confirms this finding, this means that after finishing treatment no major complications related to physiologic bone resorption are to be expected.^29^

However, there are also studies on immediate implant placement in the esthetic region, with longer follow‐up periods, showing extreme variation in buccal bone thickness, with even cases without any buccal bone. Benic and colleagues^30^ followed 14 patients over 7 years and found a median buccal bone thickness of 0.0 mm (mean 0.4 mm). Groenendijk and colleagues^31^ reported in a 2‐year retrospective study on 16 patients a buccal bone thickness of 1.8 mm (varying from 0.9 to 2.4 mm). Raes and colleagues^32^ reported on a 8‐year prospective study with 16 patients with immediate placement. Median buccal bone thickness varied from 0.80 to 1.24 mm along the implant axis and never exceeded 2 mm.

A successful esthetic treatment is dependent on realization of an optimal 3D implant position within sufficient bone dimensions and preservation of adequate bone during follow‐up, especially at the labial implant surface.^33^, ^34^ The position of the implant in relation to the labial bony wall of the alveolar ridge is thought to influence BBT after implant insertion.^35^ In the study of El Nahass and Naiem,^8^ a mean BBT of natural incisors still in situ, of 0.57 to 0.84 mm was found in the first 4 mm toward apically. Buccal bone thickness in the present study at implants is at least the BBT at natural teeth. This could mean that, probably due to the surgical procedure with palatal placement of the implant and filling the buccal space in the extraction socket between implant and buccal wall, formation and preservation of buccal bone, at least with a follow‐up of 1 year, was successful.

A limitation of analyzing buccal bone thickness on radiographs is that measuring the thickness of a radio‐opaque structure does not automatically mean that this structure is actually bone. It could well be that only limited living bone material is present in the applied bone substitute or even that only a mixture of bone substitute and connective tissues is assumed to be bone.

Clinical soft tissue outcomes, being change in midfacial mucosal level and pocket probing depth, showed limited recession in both groups and normal probing depth values in both groups. Also esthetic appearance, expressed with PES, revealed high scores without a difference between the groups. These good clinical features correspond with the presence of buccal bone at the implants in both groups, giving support to the soft tissues.

A limitation of the present study design is the direct comparison of a group with a large bony defect with a group without or with a small defect. Although the same outcome measures were used and with the same treatment team and observers, it could be that procedures between the groups differ more than just the augmentation. Next to this only a limited sample size was used; to strengthen the conclusions more patients are needed.

From this CBCT study can be concluded that:

- in patients with large bony defects at a failing tooth, it was possible to create a bone layer buccally of the implant and this bone layer remained stable during a 1‐year follow‐up;
- there were no significant differences between thickness of buccal bone at 1 month and 1 year in patients with large buccal bony defects and patients without or with small bony defects.

## CONFLICT OF INTEREST

The authors have stated explicitly that there are no conflicts of interest in connection with this article. Funding for the 1‐year clinical study was obtained by an unrestricted grant from Nobel Biocare Services AG; implant materials were provided (materials grant: 2009‐851).
